# Notes on Myotomy and Tenotomy

**Published:** 1841-09-11

**Authors:** Reynell Coates

**Affiliations:** Philadelphia


### MEDICAL EXAMINER.

DEVOTED TO MEDICINE, SURGERY, AND THE COLLATERAL SCIENCES.

No. 37.] PHILADELPHIA, SATURDAY, SEPTEMBER 11, 1841. [Vol. IV.

### ORIGINAL COMMUNICATIONS.

### NOTES ON MYOTOMY AND TENOTOMY.

BY REYNELL COATES, M. D.

To the Editors of the Medical Examiner.

Gentlemen,—Since November last, there has been some little discussion, of rather personal character, on the subject of tenotomy in cases of club-foot, in the pages of the Boston Medical Journal. The first of the series of articles directly connected with this discussion, is a paper purporting to be a "Report of cases in the Orthopedic Infirmary," and published November 25th, 1840. The second is a letter from Thomas Chadbourne, M. D., of Concord, N. H., July 17th, 1841, and published August 11th, 1841, containing a severe critique upon Dr. Brown, the author of the report. The third is an anonymous rejoinder to Dr. Chadbourne, in the number for September, 1841.

At this distance from the theatre of action, we have little interest in the temper displayed in these several papers, except such as may result from a general respect for the dignity of professional intercourse; but even this will furnish me with an ample excuse, should I venture, while discussing more important matters, to make an occasional stricture upon the mere manner of these correspondents of our excellent friend, Dr. Smith.

The report of the Infirmary contains nothing of novelty or interest to the profession, though it may possess some local importance, inasmuch as it contains a statement of the number of spinal and pedal deformities waiting for treatment at the Infirmary. The number of cases in which tendons have been already divided, is estimated by scores and hundreds, as it has been in other places, here and beyond the water; and it becomes, therefore, a serious question, how far, and under what peculiar circumstances, this fashionable operation of tendon-cutting is beneficial or injurious. I really regret that Dr. Brown has never favoured us with a more complete analysis of such a numerous range of cases as has fallen under his care—some notice of the condition of the antagonistic muscles in each case—the causes of their contraction or undue elongation—the condition of the plantar fascia, and the modifications in the form of the ancle-joint and the bones of the tarsus. But it would be unjust to censure him individually for a neglect which is common to nearly all who have written on the subject since the days of Delpech; M. Bouvier being almost the only one who has glanced over the whole field with anything like philosophical scrutiny. Another source of regret he may yet remove. Advocating, as he does, the unconditional resort to tenotomy in club-foot, he finds a long-continued after treatment of a mechanical character, indispensable for the cure.

"But time is required, and a very considerable time, for the cure of bad cases of club-foot. Bone is to be dealt with and absorbed." p. 259. "A constant pressure (not painful) must be kept up, so directed as to make a bearing upon the *external surface* of the tarsal bones." p. 260.

Waving the vagueness of these phrases, here is sufficient proof, not only that mechanical treatment is necessary in his system, which indeed is elsewhere expressly stated—but that the tenotomy is not even the most important step in the process. The mere *mechanical* division of a tendon is a much easier matter than the production of absorption in a bone. Yet the reporter for the Boston Infirmary gives us no account of *his* mechanical treatment, even while ridiculing—to use the lightest term—the exclusively mechanical treatment of club-foot by others! This is wrong. The world has a right to know the means with which he claims, very evidently, to be remarkably successful. The division of the tendons is a trifle, viewed independently of its consequences, and, like the kindred operation for strabismus, sheds little honour upon any accomplished surgeon, except its first projector: but the application of an apparatus to mould inflexible tissues—to enlarge contracted joints and modify the forms of bones, without injuring the structure or destroying the functions of the softer organs which cover them—to keep up continued pressure for a long period of time, without producing ulceration or intolerable pain—these are far nobler triumphs. There is a prevalent fashion in these modern times, which leads to the estimation of a surgeon by his ability to cut or carve with skill; and it savours much of humbug. But let me not be understood to apply this remark to any of the gentlemen whose correspondence I am canvassing. It is purely incidental. The vice is probably the result of the ridiculous regulations of the concour, as recently established in the French metropolis, where novelty rides riot over experience, and philosophers experiment against time, *or hire others to do so for them*. This folly is contagious; it has at last infected sober Germany. What wonder, then, that it should spread on this side of the water! It is not for Philadelphia to smile at Boston for running mad after new ideas, without pausing to examine or digest them. This rage for something new in surgery, has caused a retrograde progress in many of its departments, from the days of Boyer down to those of Velpeau. This novel tendon and muscle-cutting comes to us from Europe, with all the high authority of names to force it on our notice; and thus the "peer-stricken" mental dependence of our countrymen, combined with the love of easy notoriety, and sometimes—to our shame be it spoken—the emoluments received for playing on the public ignorance, which has no idea of a surgeon unattended by a knife—to give undue importance to trivial operations, and lead to their adoption to a most absurd extent. Witness the carving of the muscles of the back in curvature of the spine, the division of the tongue in stammering, &c.

Of late, the labours of a member of the profession in this city—labours unquestionably productive of curious results, though those results appear to be dependent quite as much upon unwearied care as upon any originality in the apparatus employed—have re-awakened the general attention of the profession to the readiness with which the deformities resulting from club-feet and false anchyloses, may be in great degree removed, in a vast majority of cases, by well-regulated extension, without division of any tendon, muscle, or ligament. Most of us who have been twenty years before the world, have cured club-feet in this way before any well-ordered attempt at tenotomy had been made, except, perhaps, in Delpech's single and unfortunate case. In our professional boyhood we had a pride in such efforts, and often succeeded *in a quiet way*, though, sooth to say, we never did succeed so well or constantly, for this plain reason—we had not then the untiring patience which the plan requires, and there was no hope of emolument to cheer us on—no glorious operation to serve as an advertisement!

Our friend Dr. Chadbourne seems to regard some very severe, and, I think, not unjust remarks of the reporter, as being aimed especially at this practitioner, who certainly has been exceedingly modest in the statement of results that would enhance the fame of those who have enjoyed far greater opportunities to reap distinction; and he has advanced to the defence with somewhat more of temper than *the occasion seems to require*, forgetful of the Chesterfieldian law, that every thing should be taken in a complimentary sense until we are *compelled* to regard it as insulting. I prefer extracting the whole of the passage against which he makes exception, to the selection of the two simple phrases contained in the letter.

"From time immemorial, no age or generation has been exempt from pretenders to cure spinal distortions, club-feet, &c., by mechanical means alone. These pretenders have mostly consisted of machinists, who knew little or nothing of anatomy or physiology."—It were devoutly to be wished that machinists only were thus ignorant; but, alas! for the downward tendency of medical instruction!—"Medical men have too much overlooked these complaints, until within a very short period—*either thinking them incurable, or considering them as not coming within their province for treatment;* they have quite generally recommended their patients to machine makers, who applied such apparatus as their fancy, cupidity or stupidity might suggest." Brown, loc. cit. 259.

Now all this is very true, except, perhaps, the phrase which I have ordered in italics; for Dr. B. might have found in the value of medical time, and the expense attending on the treatment of occasional cases, a more reasonable excuse for the profession. But in this very innocent passage Dr. C. discovers a gross personality. He remarks:

"Of these epithets," ('pretenders to cure club-feet,' &c.; 'machinists;' 'machine makers, who apply their own apparatus, as their fancy, cupidity, or stupidity may suggest,') "which cannot be misunderstood as intended to apply not only to Dr. Chase, but to all who advocate the opposite practice to that pursued by Dr. B., no other notice has been taken," &c.

What private knowledge Dr. Chadbourne may possess in relation to the feelings of Dr. Brown, I know not; but to the public nothing sinister is visible. Dr. Brown could not be supposed to apply terms of opprobrium to a professional brother, most of which would immediately recoil with quite equal force upon himself,—for he speaks positively in the possessive case of "*my mechanical apparatus*," and of "*instruments of my construction*," as Dr. Chadbourne expressly states when taxing him with a "studied concealment" of his mode of treatment.

Being thus avowedly as much of a "machinist" or "machine maker" as Dr. Chase, or any other honourable member of the profession who chooses to copy the example of St. Paul, and *make his own hands administer to his necessities*, instead of encountering the *cupidity and stupidity* of non-medical operatives, it is not to be supposed that he could have intended to direct these shafts at Dr. Chase, merely because the latter does not preface his mechanical treatment by the trivial operation of cutting a few tendons. The learned would smile at such a construction, were it legitimate, which it is not, and might be tempted to apply the not very dignified couplet,

> "Strange that such difference there should be
> 'Twixt tweedledum and tweedledee!"

But seriously, there seems to be so much uproar of late about the new operations among the operators themselves, that many are led to believe it a settled question among surgeons, that in all cases of club-foot, and in many of contracted knees or elbows, the division of one or more tendons is necessary to the cure. It is time that the other party spoke. Dr. Chase, who has devoted himself more particularly to these and one or two other specialties, rests contented with presenting an occasional paper containing the unvarnished narrative of his cases, without any attempt at pathological or physiological reasoning. He never generalizes, but lets facts speak for themselves; and most powerfully they do speak in favour of the use of mechanical extension independent of tenotomy; but they are not so recorded as to enable us to discriminate between the cases to which this simple treatment is adapted, and those which would be more readily and safely cured by a combination of both the knife and the machine. True, there are none—not even Dr. Chase—who would condemn the knife under all circumstances; but so far is he from standing forth as "the most able and almost only advocate" of the treatment by mechanical extention, as Dr. Chadbourne represents, that I doubt much if the majority of our well-informed surgeons approve at all of the indiscriminate cutting and slashing which seems just now to be the order of the day. That many of them do not, I know; and wishing to be ranked among this number, I shall shortly endeavour, either here or elsewhere, to give a solid reason for my faith.

If your space permit, I will request a corner in your next number for some comments on the causes that have tended to favour the idea that the treatment of club-foot by mechanical extention is too painful to be readily tolerated; and will close this note with a single remark to the anonymous correspondent of the Boston Journal. The truly scientific know no country. They are eternally at war with the influence of cliques, and the insular feelings of a village, or that congeries of villages yclept a capital. He deems the communication of Dr. Chadbourne an "exotic transplanted from the South." I do not mean to charge him with the weakness of substituting sectional feeling for argument; but the profession is a nervous profession, and exceedingly delicate in its epidermis. It is best to be in this respect like Cæsar's wife.

*Philadelphia, September* 9, 1841.

